# Geography of Food Consumption Patterns between South and North China

**DOI:** 10.3390/foods6050034

**Published:** 2017-05-05

**Authors:** Fangfang Song, Mi Sook Cho

**Affiliations:** Department of Nutritional Science and Food Management, Ewha Womans University, Daehyun-dong, Sodaemun-gu, Seoul 03760, Korea; sffkelly@126.com

**Keywords:** Chinese adults, north region, south region, dietary pattern, metabolic syndrome

## Abstract

The geographical environment, food culture, and dietary habits are substantially different between the southern and northern regions in China. We investigated the associations with dietary patterns and metabolic syndrome between Chinese adult from the southern and northern regions (North: 1249; South: 1849) using data from the Chinese Health and Nutrition 2009 survey. Respectively, four dietary patterns were identified by factor analysis in each of the two regions. Using factor analysis, each dietary pattern of factor score was calculated for three groups by tertile (T1 < T2 < T3). In the northern region, the association between the Alcohol and Western pattern and the risk of abdominal obesity (OR: 1.31; 95%: 1.01, 1.68) (OR: Odds Ratio), hypertriglyceridemia (OR: 1.35; 95%: 1.05, 1.74), high fasting blood glucose (OR: 1.37; 95%: 1.05, 1.80), and hypertension (OR: 1.55; 95%: 1.45, 1.99) was increased compared T1 to T3. In the southern region, the Convenience Food pattern was positively associated with hypertriglyceridemia (OR: 1.53; 95%: 1.03, 2.26), low high density lipoprotein (HDL)-cholesterol (OR: 1.96; 95%: 1.12, 3.43), and metabolic syndrome (OR: 1.79; 95%: 1.03, 3.11). The Alcohol dietary pattern was positively associated with high fasting blood glucose (OR: 1.83, 95%: 1.13, 2.97). There are some dietary pattern differences in the two regions. It is necessary to consider the factors of food culture and food intake habits in order to provide nutrition education to Chinese individuals from different regions in the future.

## 1. Introduction

Different dietary habits and food consumption have their own geographical distribution, especially in China [1]. Food geographers are largely taken up with retail geography as a sub-specialism within economic geography [2]. The Chinese people have developed traditional foods through their long history and also fostered a rich and diverse food culture. Due to the geographical environment and dietary habits that have formed throughout its long history, there are some dietary and cultural differences between Northern and Southern China [3]. For example, thousands of years ago, rice became a main staple food in the Southern region, while wheat was a main staple food in the North [4,5,6]. In most cultures, consumed and preferred foods are based on social and cultural influence. Although food culture changes with the passage of time and industrialization, there are still some similarities in modern regional society. Thus, it is important to consider food culture in addition to the standard nutrients and dietary pattern approach in epidemiological and geographical environment research, especially among the Chinese [7]. 

Traditional Chinese foods are mostly based on rice, wheat, and other grains [8]. In recent years, traditional foods in China have faced the increasing challenge of the incursion of Western or instant food [9,10]. In addition, following the dietary transition in China, metabolic syndrome is accelerating, and dietary intake has an intimate relation with health [11,12,13]. Dietary patterns consider the interactions between food and nutrition and represent a broader picture of food and nutrient consumption, suggesting that they may be more predictive of disease risk than individual food or nutrients [14]. In addition, dietary patterns have become a focus for nutritional research [15]. A recent factor analysis from the Chinese Health and Nutrition Survey study [16,17] identified “macho” (animal foods and alcohol), “Traditional”, “sweet tooth”(cake, milk and drinks), “vegetable-rich” dietary pattern. Traditional dietary pattern was high in rice, meat, vegetables, wheat and refined carbohydrates, and identified a “Vegetable rice” pattern, which was rich in grain, fruits, eggs, and fish as defined by factor analysis in the study. Recent studies on dietary patterns were conducted in China. Several studies [12,13] suggest that a diet rich in rice, meat, vegetables, and salted vegetables was noted as the “Traditional” pattern. Some studies [11,18] found that the “Modern High-Wheat” pattern was rich in animal products, and the high fat intake carries a high risk for metabolic syndrome, while “Vegetable rich” and “Healthy” (dairy products, eggs, fruits and vegetables) had no association with metabolic syndrome. Recent studies in China have reported [12,17,19] the relationship between dietary patterns and metabolic syndrome. However, to the best of our knowledge, no study has explored the effect of the Chinese geographical environment and food culture on the associations between dietary patterns and metabolic syndrome in Chinese adults. 

Due to the rich food culture, dietary habits are substantially different between southern and northern regions in China [4,20]. However, little is known about the relationship between dietary patterns and metabolic syndrome between the two regions. It is important to examine the diets and their association with metabolic syndrome among Chinese people based on regions. Therefore, the goal of the present study was to investigate the associations with dietary patterns and metabolic syndrome between southern and northern regions in the Chinese adult population using the Chinese Health and Nutrition 2009 survey data. 

## 2. Materials and Methods

### 2.1. Subjects

The Chinese Health and Nutrition Survey (CHNS) is an ongoing longitudinal study of nine waves (1989–2011). The samples were drawn with a multistage, random cluster process in nine provinces. The survey was conducted according to guidelines in the Declaration of Helsinki [20] and the protocols, instruments, and the process for obtaining informed consent were approved by the institutional review committees of the University of North Carolina at Chapel Hill and the Chinese Institute of Nutrition and Food Safety, China Center for Disease Control and Prevention. 

The CHNS collected blood samples for the first time in 2009; the present analysis uses the diabetes-related biomarkers measured in 2009 and the exposures, dietary intake, and covariates. Participants aged 20 years or older were included in this analysis. Information on age, gender, education, residential area, smoking, alcohol consumption, and body mass index (BMI) was collected. Participants in the study were excluded from this analysis on the basis of prevalent metabolic syndrome, metabolic syndrome risk factor variables, excessively high (>5000 kcal) or low (<500 kcal) energy intake, and missing values. We used Qinling Mountain-Huaihe River Line to divide China into north (Liaoning, Heilongjiang, Shandong, Henan) and south (Jiangsu, Hubei, Hunan, Guangxi, Guizhou) regions [21]. The final subjects consisted of 3143 (North: 1249; South: 1849) adults.

### 2.2. Macronutrient Assessment

Dietary intake was assessed via a 24-h dietary recall, assisted by a registered dietitian. Measured dietary components included energy, carbohydrates, protein, and fat. Energy was calculated using the factors of 17 kJ/g (4 kcal/g) for protein, 37 kJ/g (9 kcal/g) for fat, and 17 kJ/g (4 kcal/g) for carbohydrates. In the Chinese population, the standard proportion of total energy intake from carbohydrates was 55%–65%, 11%–15% from protein, and 20%–30% from fat [22]. The food groups included in our analysis were based on a food group classification system developed specifically for the CHNS and Chinese food composition table by researchers from the UNC-CH and INFS(12) which classifies foods according to their nutritional and behavioural significance [17,23]. Initially, 22 food groups were included. As some food item were consumed by less than 5% of participants, food intakes were further collapsed into 16 food groups in this study.

### 2.3. Extraction of Dietary Patterns

Dietary patterns were obtained by principal component factor analysis basis of 18 foods or food groups of the Chinese Food Composition [24,25]. Four dietary patterns were established between the southern and northern regions, respectively, with eigenvalue (>1) and extracted on the basis of the scree plot and evaluation of the factor loading matrix after orthogonal (varimax) rotation. Using principal component analysis, the dimension of the data was reduced by forming a few linear combinations of the original observed variables containing as much as possible of the variation in the original data. By this method, correlated variables are grouped together. The Kaiser-Meyer-Olkin (KMO) was used to measure sampling adequacy and Bartlett’s test of sphericity to assess the adequacy of test items and sample size. The coefficients defining these linear combinations, called factor loadings, are the correlations of each food item with that factor. Foods with loadings >0.4 on a factor were used to describe the dietary patterns [26]. Factor scores were created by multiplying factor loading with the corresponding standardized value for each food and summing across the food items. For each participant, the factor scores indicate the extent to which the diet conformed to the respective dietary patterns. 

### 2.4. Definition of Disease

Diabetes was defined according to the risk factors of metabolic syndrome. The metabolic syndrome was diagnosed using the updated Adult Treatment Panel III from the National Cholesterol Education Program (NCEP-ATP III) for Asian-Americans, which recognizes the existence of metabolic syndrome if three or more of the following five components are present: (1) Abdominal obesity: waist circumference ≥ 90 cm in men or ≥ 80 cm in women; (2) Hypertriglyceridemia: TG (triglyceride) ≥ 1.7 mmol/L (≥150 mg/dL); (3) Low HDL (high density lipoprotein) cholesterol: HDL-C mmol/L < 1.03 mmol/L in men or 1.3 mmol/L in women (<40 mg/dL for men, <50 mg/dL for women); (4) Hypertension: Blood pressure ≥ 130 mm Hg SBP (systolic blood pressure) or 85 mm Hg DBP (diastolic blood pressure); (5) High fasting glucose: Fasting glucose ≥ 6.1 mmol/L (≥110 mg/dL) or current drug treatment for elevated glucose. In addition, BMI was calculated as kg/m^2^. The World Health Organization has recognized the use of BMI ≥ 27.5 kg/m^2^ as a surrogate for abdominal obesity [27]. 

### 2.5. Statistical Analysis

SAS 9.4 (Statistical Analysis System, version 9.4, SAS Institute, Cary, NC, USA) was used for statistical data analysis. Analysis of variance (ANOVA) adjusted for age, gender, residential area, and energy intake was used to determine the significance of changes in total energy intake, carbohydrates, protein, and fat intakes and intake ratios among the dietary patterns. Each dietary pattern of factor scores was calculated for three groups by tertile (Low, Middle, High). For each participant the factor score indicated the extent to which her/his diet conformed to one of the dietary patterns identified. A high factor score for a given dietary pattern indicated high intake of the food groups constituting that food pattern, and a low score indicated low intake of those food groups [14,28]. There were differences in the prevalence rates of metabolic syndrome and dietary patterns using the Chi-square test. To examine the associations between dietary patterns and metabolic syndrome, multivariate nutrient density models and multivariate nutrient density substitution models were created as described in detail elsewhere [29]. Briefly, logistic regression models were constructed, using metabolic syndrome as the dependent variable. Model 1 adjusted for age, gender, residential area, income, and energy intake; model 2 further adjusted for smoking, drinking, BMI, and exercise. Logistic regression analysis was used to calculate the odds ratio (OR), and their 95% confidence interval (CI) for regression coefficients were determined. A *p*-value < 0.05 was considered statistically significant.

## 3. Results

### 3.1. Dietary Patterns

We extracted four dietary patterns by factor analysis between southern and northern regions of China, respectively (Table 1). In the northern region, the first pattern had high positive loadings on rice, tubers, and fruits, and this pattern was denoted the ‘Carbohydrate-rich’ pattern. The second pattern, which was denoted the ‘Wheat Staple’ pattern, had high positive loadings on processed wheat, vegetables, and eggs. The high-loading foods in the third pattern consisted of liquor, poultry, fungi, meat, and legumes and was denoted the ‘Alcohol and Western’ pattern. The fourth pattern had a high positive loading on convenience foods and dietary products and was denoted the ‘Convenience Food’ pattern. In the southern region, the first pattern had high positive loadings on rice, vegetables, meat, and poultry, and this pattern was denoted the ‘Traditional Southern’ pattern. The second pattern, which was denoted the ‘Convenience Food’ pattern, had high positive loadings on processed dietary products, fruits, fungi, and convenience food. The high-loading foods in the third pattern were wheat, legumes, and tubers and was denoted the ‘Carbohydrate-rich’ pattern. The fourth pattern consisted of liquor, fish, and nuts and was denoted the ‘Alcohol’ pattern.

### 3.2. The Macronutrient Intake between the South and North Regions

Each factor used a tertile that was divided into T1, T2, and T3 by factor scores. The macronutrient intake between the south and north regions are shown in Table 2. In the northern region, after adjusting for sex, age, energy intake, income, and urban/rural, in the T1 of factor 1 (Carbohydrate-rich pattern) the lowest carbohydrate intake was shown, while the T3 of the Wheat Staple pattern showed the highest energy intake. The T3 of the Alcohol and Western and Instant Food patterns showed the lowest carbohydrate intake and the highest fat intake. In the southern region, the T3 of the Traditional Southern pattern showed the highest carbohydrate and protein intake and the lowest fat intakes. The lowest carbohydrate intake and the highest protein and fat intakes were shown in the Convenience Food pattern of T3. In the Carbohydrate-rich pattern of T3 the highest carbohydrate and protein intakes and the lowest fat intake were shown. The Alcohol pattern of T3 showed the lowest carbohydrate intake and the highest protein and fat intakes. 

### 3.3. Association between Dietary Patterns and Prevalence of Metabolic Syndrome

Table 3 shows the chronic disease of metabolic syndrome according to dietary pattern. Among the northern subjects, the rate of abdominal obesity, hypertriglyceridemia, and high fasting blood glucose in the Carbohydrate-rich pattern were significantly higher in T1 than in T3. In the Wheat Staple pattern, low HDL-cholesterol was higher in T1. In the Alcohol pattern hypertriglyceridemia, high fasting blood glucose, significantly higher rates of hypertension and metabolic syndrome were shown in T3 than T1. The rates of low HDL-cholesterol, high fasting blood glucose, and metabolic syndrome among the Convenience Food pattern were significantly higher in T3 than T1. 

In the southern region, the prevalence rates of low HDL-cholesterol were significantly higher in T1 than in T3, and high fasting blood and hypertension were significantly higher in T3 among the Traditional Southern pattern, whereas in the Convenience Food pattern, the rates of low HDL-cholesterol and metabolic syndrome were significantly higher in T3 than T1. The prevalence rates of abdominal obesity, high fasting blood glucose, hypertension, and metabolic syndrome were significantly higher in T3 than T1, and in the Alcohol pattern, the rates of abdominal obesity, low HDL-cholesterol, and hypertension were significantly higher in T3 than T1.

### 3.4. The risk of Metabolic Syndrome Association with Dietary Patterns

We examined the association between dietary patterns and the risk of metabolic syndrome in the northern region (Table 4). The association between the Carbohydrate-rich pattern and abdominal obesity was decreased (OR: 0.54; 95%: 0.42, 0.69) in T3 of model 1, while the risk of hypertriglyceridemia (OR: 1.66; 95%: 1.27, 2.13) and low HDL-cholesterol (OR: 1.33; 95%: 1.01, 1.76) was increased. In the Alcohol and Western pattern, the risk of abdominal obesity (OR: 1.31; 95%: 1.01, 1.68), hypertriglyceridemia (OR: 1.35; 95%: 1.05, 1.74), high fasting blood glucose (OR: 1.37; 95%: 1.05, 1.80), and hypertension (OR: 1.55; 95%: 1.45, 1.99) were increased in T3 of model 1. In the Convenience Food pattern, the risk of hypertriglyceridemia (OR: 1.35; 95%: 1.05, 1.74) and low HDL-cholesterol (OR: 1.46; 95%: 1.09, 1.96) was increased. 

Table 5 shows the association between dietary patterns and the risk of metabolic syndrome in the southern region. The risk of metabolic syndrome in the Traditional Southern pattern showed no significant risk. The Convenience Food pattern was positively associated with hypertriglyceridemia (OR: 1.53; 95%: 1.03, 2.26), low HDL-cholesterol (OR: 1.96; 95%: 1.12, 3.43), and metabolic syndrome (OR: 1.79; 95%: 1.03, 3.11) in the model 2. The risk of high fasting blood glucose in the Carbohydrate-rich pattern was higher in T3 compared to T1 of model 1. The Alcohol dietary pattern was positively associated with high fasting blood glucose in model 1 (OR: 1.42; 95%: 1.11, 1.77) and model 2 (OR: 1.83, 95%: 1.13, 2.97).

## 4. Discussion

In this study, four dietary patterns were respectively identified by factor analysis between southern and northern Chinese adults. Northern region: Carbohydrate-rich, Wheat Staple, Alcohol and Western, and Convenience Food; Southern region: Traditional Southern, Convenience Food, Wheat, and Alcohol. Chinese people have developed traditional foods throughout their food culture, and fostered a rich and diverse food culture. Due to the geographical environment and dietary habits which formed throughout its long history, also with the economic development, there are some dietary and cultural differences between Northern and Southern China [3,4]. The food group intake between southern and northern China showed a high intake of wheat, tubers, eggs, liquor, etc. in the northern region, whereas the southern region showed a high intake of rice, vegetables, meat, poultry, fish etc. Therefore, dietary patterns that were identified in the present study are correlated with the different traditional food culture between southern and northern China and its influence on modern Chinese society. With the development of the Chinese economy in the 21st century, high intakes of instant and convenience foods and other aspects of the Western dietary pattern were also shown in China [30]. 

In this study, the Carbohydrate-rich pattern of the northern region showed a high carbohydrate diet and low fat intake. It is consistent with a study from Lee [31], which showed that a high carbohydrate dietary may lead to relatively a low fat intake. Another study [9] found that the ‘Alcohol, Nut’ pattern leads to a high fat intake, while the ‘Rice, Meat, Fish’ pattern leads to a low energy intake. These results are similar to the present study that showed a high fat intake in the Alcohol and Western pattern of the northern region. This study also showed that the Carbohydrate-rich pattern of the southern region had a high carbohydrate intake. These results are similar to a study from Liu [10], which showed that the ‘Wheat, Egg’ dietary pattern leads to a high carbohydrate and low fat intake. Other studies [32,33,34] found a high energy and fat intake in instant food or other aspects of the Western dietary pattern, which was also shown in this study. 

The Carbohydrate-rich pattern of the northern region was associated with a high risk of hypertriglyceridemia and low HDL-cholesterol. This is similar to a study from Hellerstein [35], which found that long-term high carbohydrate intake may lead to increased triglycerides. A high carbohydrate diet formed by cereal and tubers was associated with the high risk of obesity and high fasting blood glucose [16] (Shi et al., 2011). However, higher carbohydrate intake may lead to less fat intake and was associated with a lower risk of low HDL-cholesterol in this study. Other studies [36,37,38] showed that a higher alcohol intake may lead to a higher risk of abdominal obesity, hypertriglyceridemia, hypertension, and diabetes. The Korean subjects with a higher ‘Alcohol and Meat’ intake were associated with a higher risk of hypertriglyceridemia and diabetes [39]. Thus, the ‘Alcohol & Western’ pattern included a high alcohol, fat, and energy intakes in present study, and it was associated with the risk of abdominal obesity, hypertriglyceridemia, hypertension, diabetes, and metabolic syndrome [32]. Moreover, a study [40] with Korean adults who more frequently consumed instant foods showed that they had a high risk of abdominal obesity and obesity. These results are similar to the present, in which the Convenience Food pattern had a negative effect on health. 

In the southern region, the Traditional Southern pattern was not found to be associated with the risk of metabolic syndrome. Some studies [13,41] showed that rice is the staple in the Traditional Southern pattern, and because this pattern has a high intake of vegetables, fruits, eggs, fish, and meat, it is a balanced dietary pattern that may help to reduce of the risk of chronic diseases, such as abdominal obesity and hypertension. In modern society, with the development of the Chinese economy and the changes in lifestyles, there is a high intake of convenience foods among the Chinese in recent years [42]. However, because most convenience foods have high energy and fat levels, higher intakes of convenience foods may lead to a higher risk of high glucose, high triglycerides, high blood pressure, and obesity [11]. 

This study has several limitations. Firstly, the Chinese Health and Nutrition Survey data does not clearly define the diseases with low prevalence rate and is limited in identifying the risk and preventative factors of metabolic syndrome. Secondly, the statistical methods used to define the dietary patterns are somewhat subjective, including the number of factors to extract and labeling of the habits among the different regions. The present study usefully provides an example of using the food and nutrition data from the study to analyze Chinese populations from different regions. 

## 5. Conclusions

In conclusion, the development of a method of accurately evaluating an individual’s overall diet is a prerequisite for further research regarding the relationship between diets and chronic disease in China. In addition, dietary determinants of chronic disease might differ by region among Chinese. Therefore, is necessary to consider the differences of food culture and food intake between north and south China in order to provide the appropriate nutrition education among Chinese who are from different regions in the future. Practical recommendations regarding dietary guidelines for the prevention and management of chronic disease should be suggested. Nutritional intervention programs and policies should be developed by researchers and policy makers to guide and improve individual’s dietary practice associated with the risk of chronic disease. More prospective studies are required to ascertain dietary determinants which are linked to development of determinants and its components. These results in this study should also be confirmed by prospective cohorts and clinical trials in the future.

## Figures and Tables

**Table 1:** Dietary patterns in Northern and Southern subjects by factor analysis.

Food Groups	North (*n* = 1249)				South (*n* = 1849)			
	Carbohydrate-Rich	Wheat Staple	Alcohol & Western	Convenience Food	Traditional Southern	Convenience Food	Carbohydrate-Rich	Alcohol
Rice	0.866	-	-	-	0.862	-	-	-
Wheat	-	0.793	-	-	-	-	0.777	-
Tubers, starches	0.751	-	-	-	-	-	0.608	-
Legume	-	-	0.43	-	-	-	0.633	-
Vegetables	-	0.646	-	-	0.779	-	-	-
Fungi	-	-	0.57	-	-	0.488	-	-
Fruits	0.40	-	-	-	-	0.638	-	-
Nuts	-	-	-	-	-	-	-	0.545
Meats	-	-	0.54	-	0.638	-	-	-
Poultry	-	-	0.594	-	-	-	-	-
Dietary products	-	-	-	0.729	-	0.677	-	-
Egg	-	0.59	-	-	-	-	-	-
Fish	-	-	-	-	-	-	-	0.567
Fast food	-	-	-	0.813	-	0.468	-	-
Beverage	-	-	-	-	-	-	-	-
Liquor	-	-	0.627	-	-	-	-	0.764
Eigenvalue	2.83	1.71	1.64	1.63	2.99	1.86	1.81	1.55
Proportion (%)	21.75	12.32	11.74	8.76	18.67	10.81	9.12	7.72
Cumulative (%)	21.75	34.07	45.81	54.57	18.67	29.49	38.6	45.87
	(1) Factor loadings over 0.40 are shown for simplicity;				(1) Factor loadings over 0.40 are shown for simplicity;			
	(2) KMO (Kaiser-Meyer-Olkin Measure of sampling Adequacy) = 0.639;				(2) KMO (Kaiser-Meyer-Olkin Measure of sampling Adequacy) = 0.709;			
	(3) Bartlett’s Test of Sphericity Chi-square = 4165.79 (df = 91, sig. = 0.000).				(3) Bartlett’s Test of Sphericity Chi-square = 6401.70 (df = 105, sig. = 0.000).			

**Table 2:** Macronutrient intake status by dietary pattern score groups of Northern and Southern subjects.

		% of Energy			Macronutrient Intake			
		Carbohydrate (%)	Protein (%)	Fat (%)	Energy (kcal)	Carbohydrate (g)	Protein (g)	Fat (g)
North (*n* = 1249)								
Carbohydrate-rich	T1	58.16 ± 0.47 ^a^	13.61 ± 0.13 ^a^	28.22 ± 0.48 ^b^	2131.46 ± 26.66 ^NS^	305.01 ± 3.02 ^a^	71.48 ± 0.77 ^a^	69.18 ± 1.40 ^NS^
	T2	55.93 ± 0.42 ^b^	13.40 ± 0.12 ^a^	30.66 ± 0.40 ^a^	2168.22 ± 22.65 ^NS^	291.93 ± 2.45 ^b^	70.36 ± 0.58 ^a^	73.18 ± 0.97 ^NS^
	T3	57.24 ± 0.47 ^ab^	12.69 ± 0.12 ^b^	30.07 ± 0.45 ^a^	2149.5 ± 23.97 ^NS^	301.38 ± 2.52 ^a^	66.66 ± 0.57 ^b^	71.82 ± 1.01 ^NS^
	*p*-value	0.0012	<0.0001	0.0003	0.5798	0.0017	<0.0001	0.0716
Wheat staple	T1	56.44 ± 0.43 ^NS^	13.19 ± 0.12 ^NS^	30.37 ± 0.43 ^NS^	2031.9 ± 23.42 ^c^	294.77 ± 2.39	69.37 ± 0.63	73.05 ± 1.00 ^a^
	T2	57.22 ± 0.45 ^NS^	13.38 ± 0.12 ^NS^	29.40 ± 0.44 ^NS^	2175.88 ± 23.51 ^b^	301.58 ± 2.60	68.80 ± 0.62	69.60 ± 1.03 ^b^
	T3	57.68 ± 0.46 ^NS^	13.13 ± 0.12 ^NS^	29.19 ± 0.46 ^NS^	2259.61 ± 25.42 ^a^	301.96 ± 2.85	69.33 ± 0.65	71.53 ± 1.24 ^ab^
	*p*-value	0.1201	0.2501	0.1055	<0.0001	0.0562	0.8431	0.05
Alcohol & Western	T1	60.58 ± 0.46 ^a^	12.34 ± 0.11 ^c^	26.79 ± 0.49	2077.81 ± 25.65 ^c^	319.40 ± 3.03 ^a^	66.52 ± 0.66 ^b^	66.12 ± 1.49 ^c^
	T2	56.77 ± 0.41 ^b^	13.33 ± 0.13 ^b^	29.90 ± 0.40	2101.14 ± 22.85 ^bc^	300.65 ± 2.24 ^b^	70.43 ± 0.65 ^a^	71.82 ± 0.92 ^b^
	T3	53.98 ± 0.43 ^c^	13.75 ± 0.12 ^a^	32.27 ± 0.42	2270.30 ± 24.06 ^a^	278.22 ± 2.54 ^c^	71.55 ± 0.65 ^a^	76.24 ± 1.02 ^a^
	*p*-value	<0.0001	<0.0001	<0.0001	<0.0001	<0.0001	<0.0001	<0.0001
Convenience food	T1	59.46 ± 0.49 ^a^	13.13 ± 0.11 ^b^	27.41 ± 0.49 ^b^	2183.36 ± 26.48 ^a^	309.53 ± 3.20 ^a^	68.81 ± 0.68 ^b^	66.69 ± 1.40 ^b^
	T2	56.84 ± 0.42 ^b^	13.03 ± 0.13 ^b^	30.13 ± 0.41 ^a^	2076.69 ± 23.97 ^b^	297.36 ± 2.37 ^b^	68.16 ± 0.64 ^b^	72.28 ± 0.97 ^a^
	T3	55.03 ± 0.44 ^c^	13.55 ± 0.13 ^a^	31.42 ± 0.43 ^a^	2189.35 ± 23.22 ^a^	291.41 ± 2.38 ^b^	71.54 ± 0.65 ^a^	75.21 ± 1.00 ^a^
	*p*-value	<0.0001	0.0045	<0.0001	0.0009	<0.0001	0.0005	<0.0001
South (*n* = 1849)								
Traditional southern	T1	53.02 ± 0.37 ^b^	12.94 ± 0.20 ^b^	34.04 ± 0.36 ^a^	2101.45 ± 19.51 ^c^	292.08 ± 2.08 ^b^	70.18 ± 0.55 ^b^	82.85 ± 0.88 ^a^
	T2	53.18 ± 0.35 ^b^	13.25 ± 0.20 ^ab^	33.57 ± 0.35 ^a^	2231.03 ± 20.40 ^b^	290.13 ± 2.01 ^b^	70.81 ± 0.50 ^b^	81.54 ± 0.87 ^a^
	T3	55.00 ± 0.38 ^a^	13.45 ± 0.21 ^a^	31.55 ± 0.37 ^b^	2323.73 ± 20.83 ^a^	303.01 ± 2.17 ^a^	73.00 ± 0.54 ^a^	76.88 ± 0.91 ^b^
	*p*-value	0.0001	0.0014	<0.0001	<0.0001	<0.0001	0.0011	<0.0001
Convenience food	T1	56.56 ± 0.38 ^a^	12.30 ± 0.20 ^c^	31.14 ± 0.37 ^b^	2210.80 ± 23.19	310.42 ± 2.22 ^a^	65.82 ± 0.50 ^c^	74.45 ± 0.94 ^b^
	T2	52.25 ± 0.36 ^b^	13.34 ± 0.20 ^b^	34.41 ± 0.36 ^a^	2191.64 ± 18.95	287.61 ± 2.02 ^b^	72.12 ± 0.48 ^b^	84.01 ± 0.88 ^a^
	T3	52.39 ± 0.36 ^b^	14.00 ± 0.21 ^a^	33.61 ± 0.35 ^a^	2253.45 ± 19.49	287.14 ± 2.01 ^b^	76.02 ± 0.57 ^a^	82.83 ± 0.84 ^a^
	*p*-value	<0.0001	<0.0001	<0.0001	0.0681	<0.0001	<0.0001	<0.0001
Carbohydrate-rich	T1	52.68 ± 0.39 ^b^	12.94 ± 0.19 ^b^	34.38 ± 0.38 ^a^	2070.01 ± 17.95 ^c^	288.99 ± 2.21 ^b^	69.62 ± 0.51 ^b^	83.76 ± 0.91 ^a^
	T2	53.29 ± 0.34 ^b^	13.19 ± 0.20 ^b^	33.52 ± 0.33 ^a^	2174.97 ± 18.25 ^b^	292.90 ± 1.90 ^b^	71.52 ± 0.49 ^a^	81.83 ± 0.81 ^a^
	T3	55.23 ± 0.38 ^a^	13.50 ± 0.23 ^a^	31.26 ± 0.37 ^b^	2410.82 ± 23.13 ^a^	303.30 ± 2.17 ^a^	72.84 ± 0.56 ^a^	75.70 ± 0.92 ^b^
	*p*-value	<0.0001	0.0005	<0.0001	<0.0001	<0.0001	0.0001	<0.0001
Alcohol	T1	53.96 ± 0.37 ^a^	12.56 ± 0.19 ^c^	33.49 ± 0.37 ^a^	2080.56 ± 19.62 ^c^	297.85 ± 2.01 ^a^	68.18 ± 0.55 ^c^	82.03 ± 0.94 ^a^
	T2	54.97 ± 0.35 ^a^	12.96 ± 0.19 ^b^	32.06 ± 0.34 ^b^	2189.71 ± 19.17 ^b^	303.68 ± 1.90 ^a^	70.26 ± 0.45 ^b^	78.52 ± 0.83 ^b^
	T3	52.27 ± 0.38 ^b^	14.11 ± 0.22 ^a^	33.61 ± 0.38 ^a^	2385.71 ± 21.33 ^a^	283.69 ± 2.21 ^b^	75.55 ± 0.56 ^a^	80.73 ± 0.91 ^ab^
	*p*-value	<0.0001	<0.0001	0.0016	<0.0001	<0.0001	<0.0001	0.013

(1) All values are Mean ± SD (standard error of the mean) adjusted for sex, age, energy intake, income, urban/rural; (2) Each factor is divided into T1 (low percentile), T2 (middle percentile), T3 (high percentile) by factor score; (3) Means in the row on the factor groups are significantly different at the level of *p* < 0.05 by ANOVA; (4) ^a, b, c^: Means with different superscripts are significantly different by Tukey’s multiple rang test; (5) NS: No Significant.

**Table 3:** Prevalence of chronic disease by dietary patterns of Northern and Southern subjects.

Chronic Diseases		North (*n* = 1249)				South (*n* = 1849)			
		Carbohydrate-Rich	Wheat Staple	Alcohol & Western	Convenience Food	Traditional Southern	Convenience Food	Carbohydrate-Rich	Alcohol
Abdominal obesity (waist circumstances ≥ 90 cm in men, ≥80 cm in women)	T1	16.01	13.56	14.64	14.93	9.16	9.20	8.91	9.27
	T2	14.3	13.85	14.47	14.47	10.31	9.99	9.27	9.24
	T3	12.14	15.04	13.33	13.05	9.81	10.10	11.11	10.79
	*x*^2^	8.63 ***	1.3	1.25	2.37	1.29	1.02	6.20 *	3.08 *
Elevated triglycerides (serum TG ≥ 150 mg/dL)	T1	7.36	8.99	8.83	8.77	8.86	7.94	9.11	8.58
	T2	11.13	9.11	8.43	9.16	8.26	9.25	8.33	8.72
	T3	9.67	10.06	10.91	10.23	9.46	9.39	9.15	9.29
	*x^2^*	9.44 ***	0.95	4.65 **	1.56	1.68	2.82	0.88	0.60
Low HDL-cholesterol (HDL < 40 mg/dL in men, <50 mg/dL in women)	T1	7.87	10.23	8.88	6.58	8.72	6.06	8.40	8.33
	T2	8.88	8.38	8.99	9.27	8.01	8.54	8.61	8.44
	T3	8.99	7.14	7.87	9.89	6.84	8.97	6.65	6.81
	*x^2^*	0.34	6.72 **	1.08	8.71 ***	4.11 *	11.64 ***	6.06 *	3.95 *
Elevated fasting blood (plasma glucose ≥ 100 mg/dL)	T1	6.91	6.75	6.75	7.59	6.45	7.02	6.06	5.99
	T2	9.50	8.15	7.25	8.15	7.27	7.55	7.05	7.41
	T3	6.18	7.70	8.60	6.86	8.12	7.27	8.72	8.44
	*x^2^*	9.13 ***	1.55	2.82 *	1.23	3.54 *	0.36	8.93 ***	7.48 ***
Elevated blood pressure (SBP ≥ 130 mmHg or DBP ≥ 85 mmHg)	T1	7.03	7.03	6.29	7.64	3.79	6.33	3.69	4.09
	T2	8.11	6.22	7.44	8.25	6.15	5.28	6.11	5.45
	T3	6.49	8.38	7.91	5.75	7.18	5.27	7.33	7.58
	*x*^2^	3.75 *	1.54	3.91 *	3.27 *	18.43 ***	1.19	21.33 ***	19.74 ***
Metabolic syndrome	T1	6.17	6.58	5.89	6.24	4.53	3.39	4.23	4.34
	T2	8.16	6.03	6.51	7.33	4.60	4.97	4.38	4.76
	T3	5.21	6.92	7.13	5.96	5.24	5.50	5.76	5.35
	*x^2^*	6.59 **	1.18	2.67 *	3.10 *	1.24	5.39 *	5.63 *	2.24

(1) Chronic diseases prevalence (%) by Chi-square test; (2) *x^2^* significantly different at the level of *p* < 0.05. (*** *p* < 0.001, ** *p* < 0.01, * *p* < 0.05); (3) Metabolic syndrome is diagnosed by the modified NCEP ATP III criteria (any 3 of 5 constitutes, for abdominal obesity, elevated triglycerides, Low HDL-cholesterol, elevated fasting blood and elevated blood pressure). TG: triglyceride; HDL: high density lipoprotein; DBP: diastolic blood pressure.

**Table 4:** Odds ratios (and 95%) of risk factors of metabolic syndrome by dietary pattern among Northern subjects (*n* = 1249).

Chronic Diseases		Carbohydrate-Rich		Wheat Staple		Alcohol & Western		Convenience Food	
		Model 1	Model 2	Model 1	Model 2	Model 1	Model 2	Model 1	Model 2
Abdominal obesity (waist circumstances ≥ 90 cm in men, ≥80 cm in women)									
	T1	1.00	1.00	1.00	1.00	1.00	1.00	1.00	1.00
	T2	0.69 (0.54–0.88)	0.44 (0.22–0.86)	0.92 (0.72–1.17)	1.82 (0.95–3.50)	1.12 (0.88–1.42)	0.96 (0.49–1.87)	0.62 (0.48–0.80)	1.11 (0.65–1.88)
	T3	0.54 (0.42–0.69)	0.62 (0.32–1.19)	1.01 (0.79–1.30)	1.35 (0.70–2.59)	1.31 (1.01–1.68)	1.01 (0.55–1.86)	0.82 (0.48–0.80)	1.05 (0.64–1.73)
	*p* for trend	<0.0001	0.1941	0.9148	0.5106	0.0449	0.4418	0.0094	0.6256
Hypertriglyceridemia (serum TG ≥ 150 mg/dL)									
	T1	1.00	1.00	1.00	1.00	1.00	1.00	1.00	1.00
	T2	1.21 (1.01–1.72)	1.66 (1.01–2.27)	0.94 (0.73–1.23)	0.80 (0.48–1.32)	0.95 (0.73–1.23)	1.00 (0.59–1.68)	0.95 (0.73–1.23)	1.26 (0.79–2.02)
	T3	1.66 (1.27–2.13)	1.82 (1.12–2.95)	1.05 (0.80–1.37)	1.03 (0.62–1.70)	1.35 (1.05–1.74)	1.51 (0.94–2.44)	1.35 (1.05–1.74)	1.63 (1.01–2.64)
	*p* for trend	<0.0001	0.0418	0.7223	0.7978	0.0242	0.0702	0.0229	0.0462
Low HDL-cholesterol (HDL < 40 mg/dL in men, <50 mg/dL in women)									
	T1	1.00	1.00	1.00	1.00	1.00	1.00	1.00	1.00
	T2	1.20 (0.91–1.59)	1.04 (0.51–2.13)	0.82 (0.63–1.07)	0.96 (0.51–1.79)	1.05 (0.80–1.37)	0.74 (0.35–1.54)	1.33 (1.01–1.77)	1.24 (0.67–2.31)
	T3	1.33 (1.01–1.76)	2.34 (1.22–4.49)	0.72 (0.54–0.95)	0.83 (0.44–1.56)	1.04 (0.78–1.38)	1.23 (0.65–2.33)	1.46 (1.09–1.96)	1.32 (0.69–2.55)
	*p* for trend	0.0474	0.0086	0.0206	0.5454	0.8095	0.4707	0.0118	0.3798
High fasting blood (plasma glucose ≥ 100 mg/dL)									
	T1	1.00	1.00	1.00	1.00	1.00	1.00	1.00	1.00
	T2	1.36 (1.03–1.81)	1.81 (1.08–3.03)	1.13 (0.85–1.49)	1.36 (0.80–2.30)	1.10 (0.83–1.46)	1.85 (1.06–3.23)	1.17 (0.88–1.55)	2.15 (1.33–3.47)
	T3	0.74 (0.55–1.00)	0.90 (0.52–1.55)	0.98 (0.73–1.32)	0.69 (0.39–1.22)	1.37 (1.05–1.80)	1.46 (0.85–2.54)	0.90 (0.67–1.21)	1.36 (0.79–2.33)
	*p* for trend	0.3046	0.5880	0.8623	0.1090	0.0284	0.2368	0.4820	0.1376
Hypertension (SBP ≥ 130 mmHg or DBP ≥ 85 mmHg)									
	T1	1.00	1.00	1.00	1.00	1.00	1.00	1.00	1.00
	T2	1.10 (0.79–1.54)	1.71 (0.97–3.04)	0.70 (0.49–0.98)	0.90 (0.50–1.62)	0.95 (0.73–1.23)	1.00 (0.59–1.68)	1.54 (1.11–2.14)	1.42 (0.84–2.43)
	T3	1.03 (0.72–1.46)	1.38 (0.75–2.52)	0.79 (0.57–1.10)	1.08 (0.60–1.93)	1.35 (1.05–1.74)	1.51 (0.94–2.44)	1.00 (0.70–1.45)	0.94 (0.52–1.69)
	*p* for trend	0.8648	0.3064	0.2095	0.7196	0.9004	0.9690	0.8536	0.9969
Metabolic syndrome									
	T1	1.00	1.00	1.00	1.00	1.00	1.00	1.00	1.00
	T2	1.46 (1.06–2.02)	1.85 (0.95–3.61)	0.81 (0.57–1.13)	0.53 (0.24–1.15)	1.15 (0.83–1.59)	0.89 (0.41–1.93)	1.50 (1.08–2.08)	1.45 (0.76–2.78)
	T3	1.02 (0.72–1.45)	1.25 (0.60–2.59)	0.80 (0.57–1.12)	0.86 (0.42–1.78)	1.45 (1.05–1.99)	1.09 (0.57–2.08)	1.24 (0.88–1.75)	1.21 (0.62–2.37)
	*p* for trend	0.8374	0.5174	0.1979	0.9920	0.0338	0.7553	0.1873	0.4914

(1) Model 1: Adjusted for age, gender, urban/rural, income and energy intake; (2) Model 2: Adjusted for age, gender, urban/rural, income, energy intake, smoking, drinking, BMI and exercise; (3) OR(95% CI), ORs from the Medium, High relative to the Low; (4) Metabolic syndrome is diagnosed by the modified NCEP ATP III criteria (any 3 of 5 constitutes, for abdominal obesity, elevated triglycerides, Low HDL-cholesterol, elevated fasting blood and elevated blood pressure).

**Table 5:** Odds ratios (and 95%) of risk factors of metabolic syndrome by dietary pattern among Southern subjects (*n* = 1849).

Chronic Diseases		Traditional Southern		Convenience Food		Carbohydrate-Rich		Alcohol	
		Model 1	Model 2	Model 1	Model 2	Model 1	Model 2	Model 1	Model 2
Abdominal obesity (waist circumstances ≥ 90 cm in men, ≥80 cm in women)									
	T1	1.00	1.00	1.00	1.00	1.00	1.00	1.00	1.00
	T2	0.92 (0.74–1.36)	1.09 (0.62–1.91)	1.07 (0.87–1.33)	1.15 (0.67–1.99)	0.91 (0.74–1.13)	0.91 (0.52–1.58)	0.82 (0.67–1.02)	0.58 (0.33–1.03)
	T3	0.83 (0.67–1.03)	0.60 (0.34–1.05)	0.90 (0.73–1.12)	0.53 (0.30–0.92)	1.07 (0.86–1.32)	0.73 (0.41–1.30)	0.93 (0.75–1.15)	0.69 (0.39–1.23)
	*p* for trend	0.0937	0.0558	0.3477	0.0403	0.5549	0.2664	0.5153	0.2650
Hypertriglyceridemia (serum TG ≥ 150 mg/dL)									
	T1	1.00	1.00	1.00	1.00	1.00	1.00	1.00	1.00
	T2	0.88 (0.72–1.09)	0.86 (0.57–1.30)	1.31 (1.06–1.61)	1.20 (0.82–1.76)	0.86 (0.70–1.06)	0.97 (0.65–1.45)	1.00 (0.81–1.23)	0.82 (0.53–1.27)
	T3	1.01 (0.82–1.24)	1.13 (0.78–1.65)	1.41 (1.13–1.75)	1.53 (1.03–2.26)	0.92 (0.74–1.14)	1.06 (0.72–1.56)	1.04 (0.84–1.29)	1.18 (0.80–1.74)
	*p* for trend	0.9350	0.4468	0.0022	0.0366	0.4342	0.7583	0.6907	0.3287
Low HDL-cholesterol (HDL < 40 mg/dL in men, <50 mg/dL in women)									
	T1	1.00	1.00	1.00	1.00	1.00	1.00	1.00	1.00
	T2	0.84 (0.67–1.05)	0.90 (0.51–1.58)	1.33 (1.06–1.68)	1.27 (0.73–2.20)	1.06 (0.85–1.32)	1.43 (0.82–2.47)	1.04 (0.83–1.30)	0.94 (0.51–1.71)
	T3	0.89 (0.70–1.12)	0.89 (0.53–1.52)	1.61 (0.91–1.48)	1.96 (1.12–3.43)	0.87 (0.68–1.12)	1.01 (0.56–1.83)	0.87 (0.69–1.11)	1.16 (0.66–2.05)
	*p* for trend	0.3038	0.6842	0.2539	0.0201	0.2868	0.9649	0.2800	0.5726
High fasting blood (plasma glucose ≥ 100 mg/dL)									
	T1	1.00	1.00	1.00	1.00	1.00	1.00	1.00	1.00
	T2	1.08 (0.85–1.36)	1.18 (0.73–1.89)	1.15 (0.92–1.44)	1.32 (0.87–2.00)	1.14 (0.90–1.43)	1.26 (0.78–2.02)	1.23 (0.98–1.55)	1.60 (0.96–2.65)
	T3	1.18 (0.94–1.48)	1.41 (0.90–2.19)	1.12 (0.89–1.42)	1.19 (0.76–1.84)	1.40 (1.11–1.77)	1.42 (0.90–2.24)	1.42 (1.11–1.77)	1.83 (1.13–2.97)
	*p* for trend	0.1595	0.1248	0.3350	0.3901	0.0046	0.1378	0.0053	0.0158
Hypertension (SBP ≥ 130 mmHg or DBP ≥ 85 mmHg)									
	T1	1.00	1.00	1.00	1.00	1.00	1.00	1.00	1.00
	T2	1.20 (O0.89–1.04)	0.95 (0.53–1.72)	1.00 (0.76–1.31)	0.71 (0.45–1.12)	1.38 (1.02–1.63)	1.25 (0.78–2.01)	1.03 (0.77–1.39)	0.83 (0.47–1.44)
	T3	0.90 (0.69–1.18)	1.11 (0.64–1.92)	0.88 (0.66–1.68)	0.82 (0.51–1.33)	1.21 (0.89–1.63)	0.99 (0.61–1.60)	1.24 (0.93–1.65)	1.32 (0.82–2.13)
	*p* for trend	0.2299	0.8476	0.3694	0.3837	0.3065	0.8389	0.1247	0.1519
Metabolic syndrome									
	T1	1.00	1.00	1.00	1.00	1.00	1.00	1.00	1.00
	T2	0.78 (0.59–1.04)	0.95 (0.53–1.72)	1.36 (1.02–1.80)	1.27 (0.74–2.18)	0.90 (0.68–1.20)	1.47 (0.82–2.64)	0.91 (0.69–1.20)	1.01 (0.53–1.92)
	T3	0.90 (0.69–1.18)	1.11 (0.64–1.92)	1.37 (1.02–1.83)	1.79 (1.03–3.11)	1.11 (0.84–1.46)	1.22 (0.68–2.18)	0.97 (0.74–1.28)	1.30 (0.75–2.26)
	*p* for trend	0.5096	0.6652	0.0396	0.0398	0.4345	0.6235	0.8744	0.3063

(1) Model 1: Adjusted for age, gender, urban/rural, income and energy intake; (2) Model 2: Adjusted for age, gender, urban/rural, income, energy intake, smoking, drinking, BMI and exercise; (3) OR (95% CI), ORs from the Medium, High relative to the Low; (4) Metabolic syndrome is diagnosed by the modified NCEP ATP III criteria (any 3 of 5 constitutes, for abdominal obesity, elevated triglycerides, Low HDL-cholesterol, elevated fasting blood and elevated blood pressure).
